# CD44+/EPCAM+ cells detect a subpopulation of ALDH^high^ cells in human non-small cell lung cancer: A chance for targeting cancer stem cells?

**DOI:** 10.18632/oncotarget.27568

**Published:** 2020-04-28

**Authors:** Valentina Masciale, Giulia Grisendi, Federico Banchelli, Roberto D’Amico, Antonino Maiorana, Pamela Sighinolfi, Alessandro Stefani, Uliano Morandi, Massimo Dominici, Beatrice Aramini

**Affiliations:** ^1^Division of Thoracic Surgery, Department of Medical and Surgical Sciences, University of Modena and Reggio Emilia, Modena, Italy; ^2^Division of Oncology, Department of Medical and Surgical Sciences, University of Modena and Reggio Emilia, Modena, Italy; ^3^Center of Statistic, Department of Medical and Surgical Sciences, University of Modena and Reggio Emilia, Modena, Italy; ^4^Institute of Pathology, Department of Medical and Surgical Sciences, University of Modena and Reggio Emilia, Modena, Italy; ^*^Co-first/last authors

**Keywords:** immunophenotype, cancer stem cells, cancer stem-like cells, non-small cell lung cancer (NSCLC), target therapy

## Abstract

Objectives: Several studies demonstrated that aldehyde dehydrogenase (ALDH) and CD44 are the most considered cancer stem cells (CSC) markers. However, a comparison between ALDH high cells and CD44+ cells have been previously described with no significant correlation. Indeed, the aim of the present research is to identify a superficial marker able to match with ALDH high cells population in freshly isolated human lung cancer cells.

Materials and Methods: This cross-sectional study analyzed the expression of ALDH^high/low^ cells and the positivity for CD44 and epithelium cell adhesion molecule (EPCAM) antigens in surgical lung cancer tissues. The main approach was a cytofluorimetric analysis of ALDH expression and positivity for CD44/EPCAM on primary cell population obtained from 23 patients harboring NSCLC.

Results: There was a highly positive correlation between the expressions of ALDH^high^ and CD44+/EPCAM+ cells, with a Pearson’s correlation coefficient equal to 0.69 (95% CI 0.39–0.86; *P* = 0.0002), and Spearman’s correlation coefficient equal to 0.52 (*P* = 0.0124). The average paired difference between the expression of ALDH^high^ and CD44+/EPCAM+ cells was very close to 0, being 0.1% (SD 2.5%); there was no difference between these subpopulations in terms of means (95% CI = –1.0; 1.2%, *P* = 0.8464). These results highlight a strong similarity between ALDH^high^ and CD44+/EPCAM+ cells.

Conclusions: Our study is the first attempt which identifies a high correlation between the ALDH^high^ and the CD44+/EPCAM+ cells, thus suggesting the possibility to use this superficial marker for future target treatments against lung cancer stem cells.

## INTRODUCTION

The cancer stem cell (CSC) model was proposed over 30 years ago [1] and is a very important field of study in cancer research. The frequency of CSCs varies from 27% to 100% in highly tumorigenic cancers, such as hematopoietic and melanoma primary tumors, as well as in some cancer cell lines [2]. Although CSCs account for less than 1% of the total cells in solid tumors [3], they have several roles in tumor generation and progression, such as in the capacity for self-renewal, asymmetric cell division, slow division kinetics, increased capacity of invasion, metastasis, tumor formation, proliferation, resistance to conventional chemotherapy, and radiotherapy and immunotherapy [4–7]. For the small amount of these cells inside the tumor, however, several studies have been conducted on the identification of CSC markers [8, 9].

The most considered CSC markers have been aldehyde dehydrogenase (ALDH), and CD44+ [10]. In particular, ALDH was described in 2010 by Sullivan et al. as a cancer stem cell marker in a panel of 11 non-small-cell lung cancer (NSCLC) tumor samples, 45 NSCLC lines, and 7 small-cell lung cancer (SCLC) lines [11]. Additionally, this small population of CSCs was better identified using multiple markers [12]. In particular, Wang et al. in 2013 established a panel of lung cancer cell lines from primary tumors and characterized a small subpopulation as strongly positive for CD44 (CD44^high^), with the main population being weakly positive or negative for CD44. Co-expression of CD90 (CD90^+^) further narrowed down the putative stem cell population. This CD44 and CD90 positive subpopulation showed several CSC characteristics. In fact, the CD44^high^ CD90^+^ subpopulation was considered a good candidate for a CSC marker [4].

In 2015, N. Zakaria et al. showed the putative lung CSC phenotypes of CD166+/CD44+ and CD166+/EPCAM+ with multipotent characteristics of stem cells in lung adenocarcinoma cells (A549 and H2170) [8].

The identification of multiple markers is due to the complexity of highlighting the entire cancer stem cell population. Hence, a triple-positive marker, EPCAM+/CD166+/CD44+, has recently been described in the human non-small cell lung cancer cell line [12].

Nevertheless, although ALDH is considered an intracellular enzyme and is the most used marker to identify CSCs in lung cancer [7, 11, 13], the scientific community has never correlated this intracellular marker with an epithelial marker, which may be very useful for targeting lung cancer stem cells. The aim of the present research was to compare the ALDH^high^ cells with the double-positive CD44/EPCAM cells, in order to better define the superficial antigens of ALDH^high^ cells. The use of superficial antigens may trigger new therapeutic approaches against CSCs in NSCLC.

## RESULTS

### Patients’ and specimens’ characteristics

The major clinical and demographic characteristics of the 24 patients enrolled from December 2017 to January 2019 are reported in Table 1. The average age was 70.3 years (SD 9.3, range 52 to 84); 62.5% were males, and all were smokers. Patients harboring stage I (37.5%) and stage III (37.5%) NSCLC were more prevalent than those harboring stage II (25.0%), whereas adenocarcinoma (75.0%) was more frequent than squamous cell carcinoma (25.0%). The surgical tumor specimens’ average weight was 1.3 grams (SD 1.9, range 0.1 to 9.6), and the average cellular yield was 33.8 million cells per gram (SD 35.9, range 7 to 150).

**Table 1:** Descriptive characteristics of the patients and specimens included in the study

Characteristics of patients		All patients (*n* = 24)
Age (years)	mean ± SD	70.3 ± 9.3
	median (range)	70 (52; 84)
Sex – M	*n* (%)	15 (62.5%)
Smoker – Yes	*n* (%)	24 (100.0%)
Adenocarcinoma	*n* (%)	18 (75.0%)
Squamous cell carcinoma	*n* (%)	6 (25.0%)
Stage - I	*n* (%)	9 (37.5%)
Stage - II	*n* (%)	6 (25.0%)
Stage - III	*n* (%)	9 (37.5%)
Characteristics of specimens		All samples (*n* = 24)
Weight (grams)	mean ± SD	1.3 ± 1.9
	median (range)	0.8 (0.1; 9.6)
Cellular yield (million cells per gram)	mean ± SD	33.8 ± 35.9
	median (range)	19.7 (7.0; 150.0)
Cytofluorimetric analysis		All samples (*n* = 23)
ALDH+ (% on 7AAD- cells)	mean ± SD	3.2 ± 3.4%
	median (range)	1.9% (0.4; 12.5%)
CD44+/EPCAM+ (% on 7AAD- cells)	mean ± SD	3.1 ± 2.5%
	median (range)	2.6% (0.1; 10.1%)
CD44+/EPCAM– (% on 7AAD- cells)	mean ± SD	11.7 ± 22.9%
	median (range)	2.5% (0.2; 86.0%)
CD44–/EPCAM+ (% on 7AAD- cells)	mean ± SD	18.5 ± 19.4%
	median (range)	12.5% (0.0; 64.0%)

### Cytofluorimetric analysis of ALDH^high^ and CD44+/EPCAM+ in primary lung cancer cells

**Figure 1:**
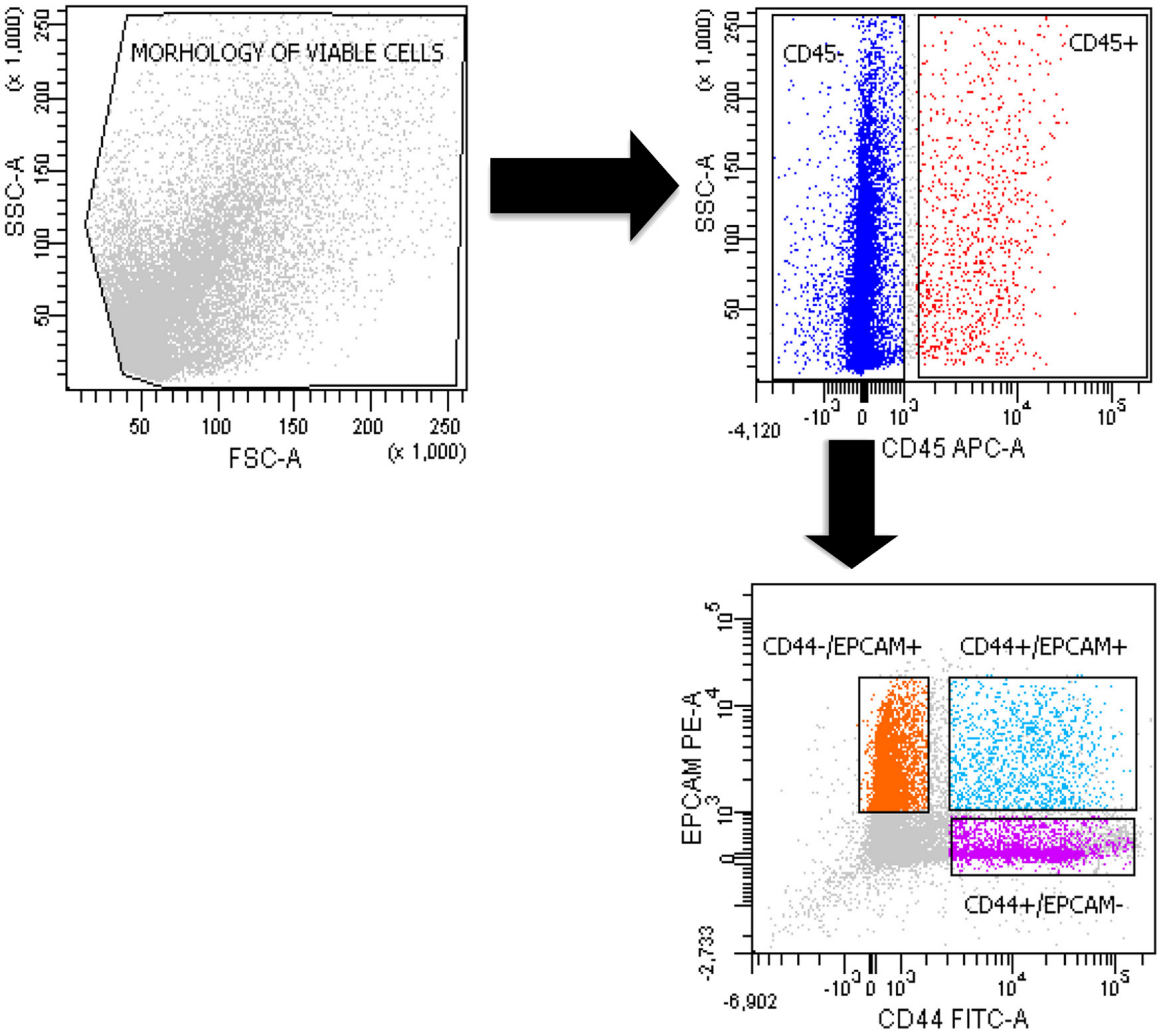
Sorting of double-positive CD44+/EPCAM+ and negative CD44+/EPCAM– and CD44–/EPCAM+ cells. The gating strategy of a representative FACS analysis of a primary tumor cell suspension in 1 patient. We used 7-AAD to detect live cells and CD45 to exclude the hematopoietic cell populations.

The putative CSCs were physically separated from the bulk parental tumor cells and recovered by fluorescence-activated cell sorting (FACS) according to the following gating strategy. Tumor cells were first identified based on their morphological parameters (forward scatter versus side scatter (FSC/SSC)), and ALDH activity was measured in the 7-AAD-negative cell subpopulation only. ALDH^low^ and ALDH^high^ cells were selected and sorted. Results obtained in the main cytofluorimetric analysis are reported in Table 1. An ALDH^high^ subpopulation was identified for all patients, and the average expression was 3.2% (SD 3.4%, range 0.4% to 12.5%, with 69.6% of samples above 1%). A CD44+/EPCAM+ subpopulation was also identified in all samples, exhibiting an average expression equal to 3.1% (SD 2.5%, range 0.1% to 10.1%, with 82.6% of samples above 1%), similar to that of ALDH^high^ cells (Figure 1). We also identified CD44+/EPCAM– and CD44–/EPCAM+ subpopulations with average expressions equal to 11.7% and 18.5% (SD 22.9% and 19.4%), respectively (Table 1) (Figure 1). The present results did not relevantly change if considering only the subgroup of 18 patients harboring adenocarcinoma (Supplementary Table 1, Supplementary Figure 1).

### Relationship between ALDH^high^ cancer stem-like cells and CD44+/EPCAM+ cells in primary lung cancer

As per the data reported in Table 2, there was a highly positive correlation between the expression of ALDH^high^ cells and the expression of CD44+/EPCAM+ cells, with a Pearson’s linear correlation coefficient equal to 0.69 (95% CI 0.39–0.86; *P* = 0.0002), and Spearman’s rank correlation coefficient was equal to 0.52 (*P* = 0.0124). Conversely, no correlation was observed between ALDH^high^ and CD44+/EPCAM– cell subpopulations or between ALDH^high^ and CD44–/EPCAM+ cell subpopulations (Table 2).

**Table 2:** Correlation of ALDH^high^ cells expression with expression of CD44+ and EPCAM+ cells

Antigens	Pearson’s correlation		Spearman’s correlation	
	r (95% CI)	*p*	r	*p*
CD44+/EPCAM+	0.69 (0.39; 0.86)	0.0002	0.52	0.0124
CD44+/EPCAM–	0.07 (–0.35; 0.48)	0.7429	0.19	0.3918
CD44–/EPCAM+	–0.29 (–0.63; 0.14)	0.1810	-0.30	0.1663

r = correlation coefficient; 95% CI = 95% confidence interval; *p* = *p*-value.

The results of the assessment of the differences in the expression of ALDH^high^ and CD44+/EPCAM+ cells are reported in Table 3. The average paired difference between the expression of ALDH^high^ and CD44+/EPCAM+ cells was very close to 0, being 0.1% (SD 2.5%, range –5.2% to 5.0%). Moreover, 43.5% of samples had a difference of less than 1% between the expression of ALDH^high^ cells and the expression of CD44+/EPCAM+ cells, and 69.6% of samples had a difference of less than 2% between the expression of ALDH^high^ cells and the expression of CD44+/EPCAM+ cells. Based on the paired *t*-test, there was no difference between these subpopulations in terms of means (*P* = 0.8464), and the 95% confidence interval for the paired difference was very narrow, ranging from –1.0% to 1.2%. Considering a target difference equal to 2% and based on the observed standard deviation for the difference between ALDH^high^ and CD44+/EPCAM+ cells, the power of the paired *t*-test would be equal to 95.9%; if considering a target difference of 1.5% or 1%, the corresponding powers would be equal to 79.2% and 45.6%, respectively.

**Table 3:** Analysis of equality of expressions of ALDH^high^ and CD44+/EPCAM+ cells

		All samples (*n* = 23)	*p*-value
Δ (ALDH^high^ minus CD44+/EPCAM+)	mean ± SD (95% CI)	0.1 ± 2.5% (–1.0 ; 1.2%)	0.8464
	median (range)	0.0% (–5.2 ; 5.0%)	
|Δ| ≤ 1%	*n* (%) (cum %)	10 (43.5%) (43.5%)	
|Δ| ≤ 2.5%	*n* (%) (cum %)	6 (26.1%) (69.6%)	
|Δ| ≤ 5%	*n* (%) (cum %)	5 (21.7%) (91.3%)	
|Δ| ≤ 7.5%	n (%) (cum %)	2 (8.7%) (100.0%)	

SD = standard deviation; Δ = difference between ALDH+ and CD44+/EPCAM+ expression; 95%CI = 95% confidence interval; cum % = cumulative percentage.

**Figure 2:**
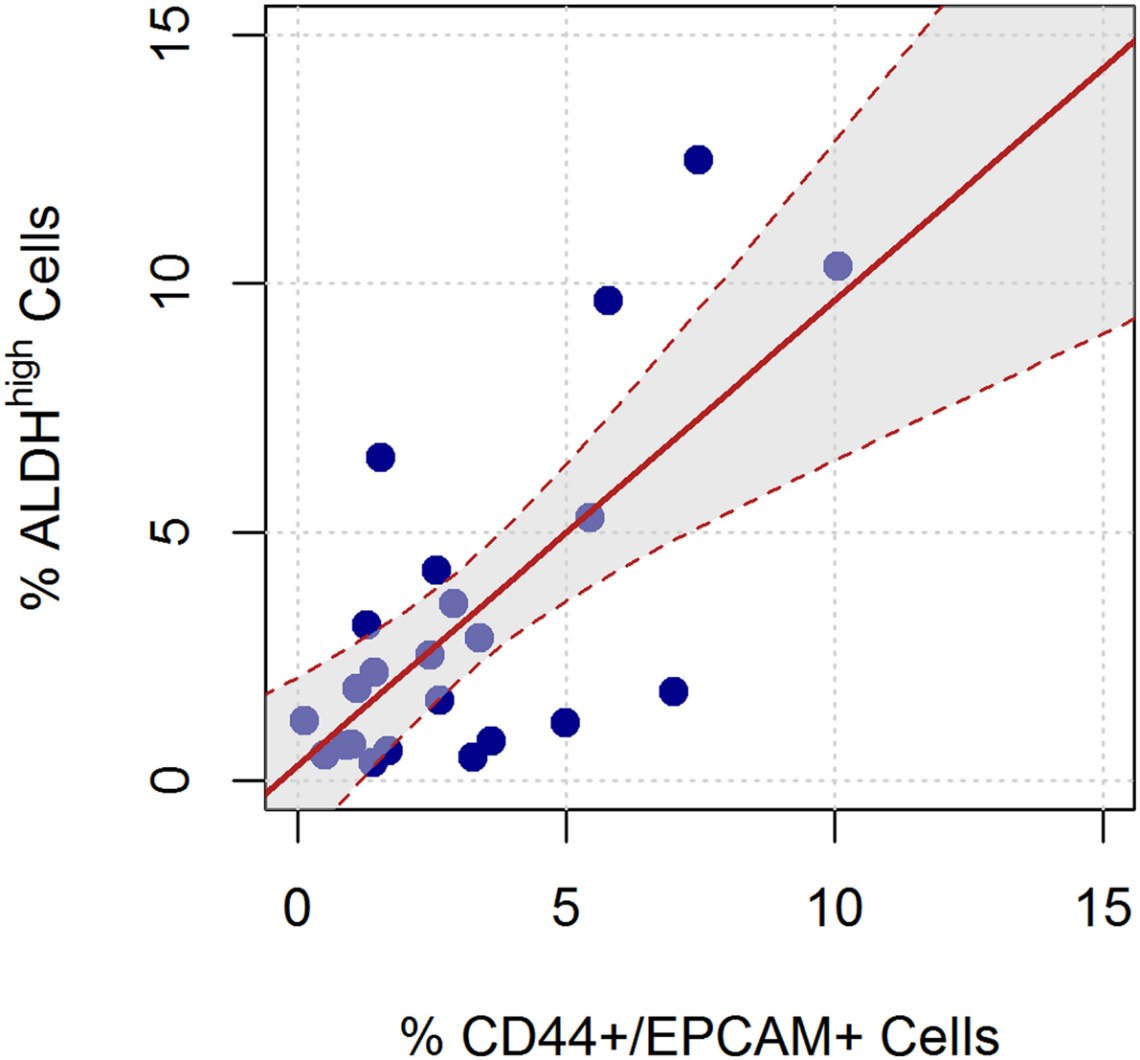
Linear relation between the ALDH^high^ and CD44+/EPCAM+ sorted cells. The red line represents the linear regression equation, considering ALDH^high^ as the dependent variable and CD44+/EPCAM+ as the independent variable. The shaded area represents the confidence interval for the regression equation.

Finally, based on the linear regression model, we estimated that a 1% increase in CD44+/EPCAM+ expression yields on average a 0.9% increase in ALDH+ expression (95% CI 0.5%–1.3%). The estimated regression equation was very similar to the line representing equality in the expression of the 2 types of cells (Figure 2).

### Cytofluorimetric analysis of ALDH ^high/low^ sorted cell subpopulations for the identification of CD44+/EPCAM+ cells

**Figure 3:**
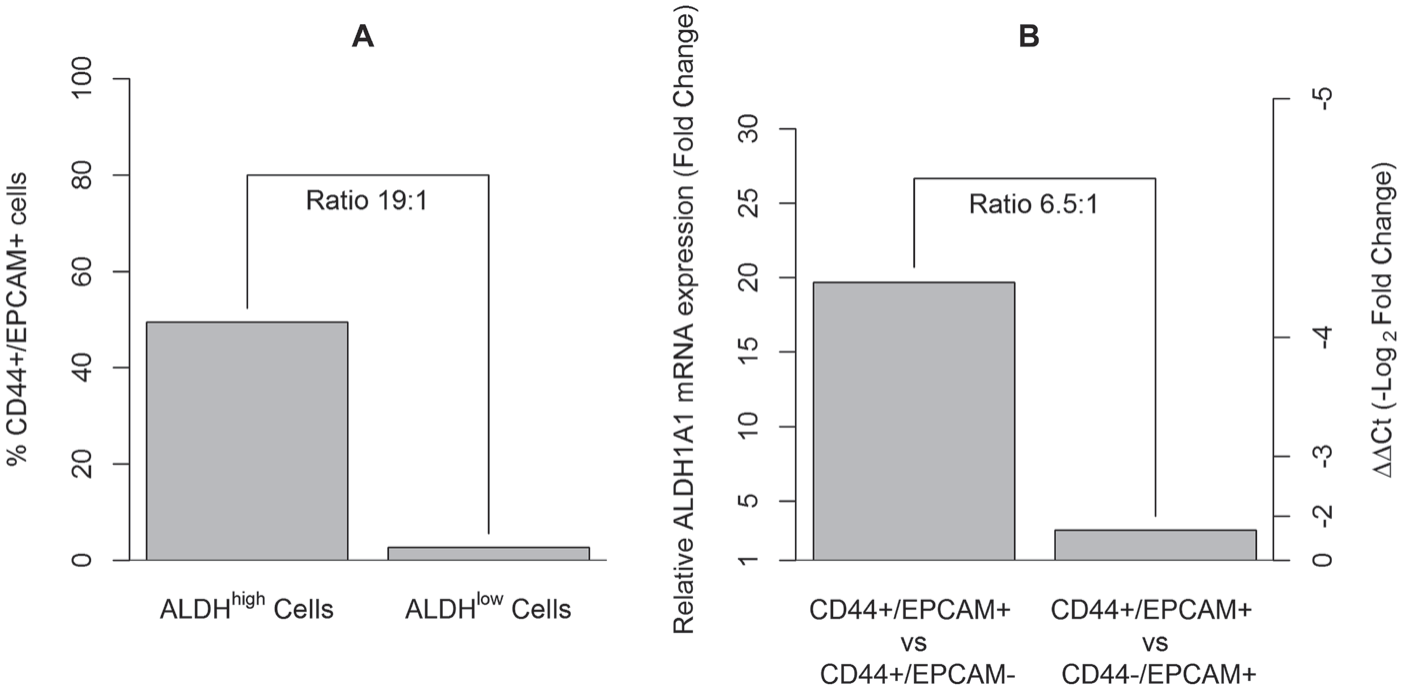
The assessment of CD44+/EPCAM+ cells in ALDH^high^ sorted cells and their enrichment for the ALDH1A1 cancer stem cell gene. (**A**) Approximately 50% of the ALDH^high^ sorted cells were CD44+/EPCAM+ cells, whereas only 2.6% of the the ALDH^low^ cells were CD44+/EPCAM+ cells. (**B**) Gene expression analysis of ALDH1A1 assessed in CD44+/EPCAM+, CD44+/EPCAM–, and CD44–/EPCAM+ sorted cells. Histogram on the left axis showed a fold change equal to 19.3 times (ΔΔCt = –4.3) when comparing CD44+/EPCAM+ to CD44+/EPCAM– cells and a fold change equal to 3.0 times (ΔΔCt = –1.6) when comparing CD44+/EPCAM+ to CD44–/EPCAM+ cells.

Primary lung cancer cells derived from a 65-year-old male patient who had undergone surgery for stage IIB NSCLC were sorted by FACS to isolate ALDH^high^ and ALDH^low^ cells. Subsequently, both ALDH^high^ and ALDH^low^ cells were further analyzed for CD44+/EPCAM+ expression by FACS, setting a gate on 7AAD- and CD45. The results showed that a total of 49.4% of ALDH^high^ cells were CD44+/EPCAM+, whereas only 2.6% of ALDH^low^ cells were CD44+/EPCAM+ (ratio 19:1) (Figure 3A).

### CD44+/EPCAM+ sorted cells have the ability to form tumor spheres *in vitro*

**Figure 4:**
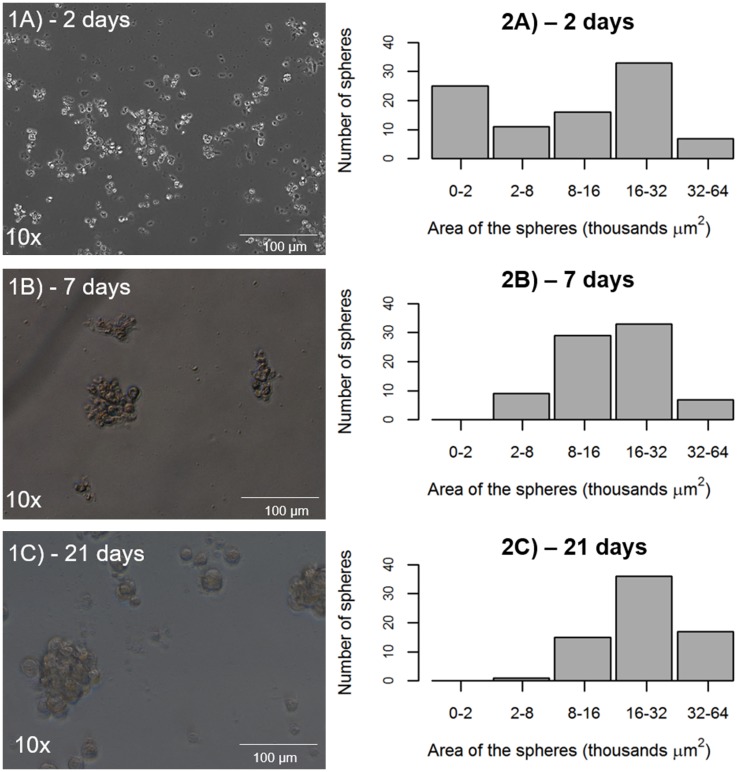
Potential of tumor sphere formation by CD44+/EPCAM+ sorted cells. (**1A**–**1C**) representative tumor spheres from CD44+/EPCAM+ sorted cells at 2, 7, and 21 days. (**2A**–**2C**), tumor spheres exhibit the same number over time and increased area, as measurement at 2, 7, and 21 days indicates.

Primary sorted CD44+/EPCAM+ cells derived from a 74-year-old male patient, who underwent surgery for stage IIIA NSCLC, were cultured to assess the growth ability of these cells to form tumor spheres *in vitro* (Figure 4, Panel 1A–1C). At the time point of 2 days after the seeding, the CD44+/EPCAM+ cells had formed non-adherent spheres in culture, and more than 50% of these tumor spheres were either small or medium in size, with a total area below 16.000 μm^2^. At the time point of 7 days after the seeding, there were no more spheres of 20.000 μm^2^ or below in area, and the spheres belonging to the other classes had not increased in number since the time point of 2 days after the seeding. At the end point, 21 days after the seeding of the cells, only the tumor spheres of medium/large area remained, with spheres belonging to the classes of 16.000–32.000 μm^2^ in area and 32.000-64.000 μm^2^ in area representing 50% of the total spheres in culture (Figure 4, Panel 2A–2C).

### Gene expression of ALDH1A1 cancer stem cell marker according to positivity for CD44/EPCAM

As an additional experiment, primary cells derived from a 76-year-old male patient, who underwent surgery for stage IA2 NSCLC, were co-stained for anti-CD44 and anti-EPCAM antibodies for selection by cell sorting. The gene expression of ALDH1A1 was assessed in the CD44+/EPCAM+ cells in comparison with the other cell subpopulations sorted, the CD44+/EPCAM– cells and the CD44–/EPCAM+ cells. A fold change equal to 19.3 times (ΔΔCt = –4.3) was found when comparing CD44+/EPCAM+ cells to CD44+/EPCAM– cells, whereas a fold change equal to 3.0 times (ΔΔCt = –1.6) was observed when comparing CD44+/EPCAM+ cells to CD44–/EPCAM+ cells. The ratio of these two fold changes was approximately 6.5:1 (Figure 3B).

## DISCUSSION

Lung cancer has remained the most commonly occurring cancer globally. Traditional methods such as surgery, radiotherapy, and chemotherapy are treatment methods for lung cancer. Resistance to radiotherapy and chemotherapy and cancer relapse, however, remain challenging issues in lung cancer treatment. Chemoresistance is a major problem in the treatment of cancer patients, as cancer cells become resistant to the chemical substances used in treatment, thereby limiting the efficiency of chemo agents [14]. This resistance is attributable to a class of cells known as cancer stem cells. In fact, accumulating evidence suggests that the cancer stem cell population is responsible for chemoresistance and cancer relapse, as CSCs have the ability to self-renew and to differentiate into heterogeneous lineages of cancer cells in response to chemotherapeutic agents [15, 16]. CSCs are also able to induce cell cycle arrest (quiescent state), which supports their ability to become resistant to chemotherapy and radiotherapy [17, 18].

Although successful cancer therapy abolishes the bulk of proliferating tumor cells, a subset of remaining CSCs can survive and promote cancer relapse due to their ability to establish higher invasiveness and chemoresistance [18, 19]. Understanding the features of CSCs will be important in establishing the foundation for a new era in the treatment of cancer. To achieve this goal, we strongly believe that an accurate identification of CSC antigens may be helpful in improving knowledge of this small but harmful population. This is why we conducted our experiments comparing ALDH^high^ and CD44+/EPCAM+ cell subpopulations.

The choice to use these markers derived from recent studies demonstrating that ALDH and CD44 are the most common CSC markers in many solid tumors [18, 20]. Nevertheless, in our previous study [15], we found comparable expression of ALDH^high^ and CD44+ cells without a significant positive correlation, hence the necessity to add another antigen for identifying the cells that are the most similar to ALDH^high^ cells, which are actually considered cancer stem cells [21, 22]. The choice to use EPCAM derived from a specific characteristic of this marker: it is an epithelial cell adhesion molecule, expressed by solid tumors of epithelial origin, such as non-small-cell lung cancer, breast cancer, or ovarian cancer [23]. Moreover, it is well recognized that EPCAM positive cells possess tumor-initiating potential, and EPCAM has already been used as a key marker of ovarian cancer stem cells [24]. These findings strongly support the notion that EPCAM is an ideal therapeutic target for ovarian cancer. In fact, various EPCAM antagonists have been developed as EPCAM-targeted antibodies serving as an effective treatment in both experimental models and clinical trials. From these findings, we decided to use CD44 together with EPCAM, comparing these selected cell subpopulations with the ALDH^high^ cell subpopulation.

Interestingly, we found that there is a highly positive linear correlation between ALDH^high^ cells and CD44+/EPCAM+ cells. Furthermore, a very strong similarity between these cell subpopulations in terms of means was found, and their linear relationship was assessed as very close to equality. Conversely, we observed no correlation between ALDH^high^ and CD44+/EPCAM– cells or between ALDH^high^ and CD44–/EPCAM+ cells. These results suggested to us that the closest cells to ALDH^high^ cells are CD44+/EPCAM+ cells. We regard this as a relevant result, since to the authors’ knowledge this is the first study that investigated and identified a robust correlation between these populations of cancer stem cells. Based on our observed results, this is the first study, which demonstrates that these two cancer stem cells populations seem to match closely with each other.

As performed previously [12], we decided to assess the tumor sphere formation ability of CD44+/EPCAM+ cells. We observed a growth of tumor spheres for up to 3 weeks, with results that were similar to those previously recorded for ALDH^high^ cells in terms of the number and size of spheres [7].

As proof of concept of the ALDH enrichment in the population identified by CD44+/EPCAM+ cells, we performed an additional experiment in which primary lung cancer cells were sorted for CD44/EPCAM antigens. The ALDH mRNA expression was evaluated by real-time PCR in CD44+/EPCAM+, CD44+/EPCAM–, and CD44–/EPCAM+ cell subpopulations. Our results showed an abundant overexpression of the ALDH1A1 gene (fold change of 19.3) for CD44+/EPCAM+ cells versus CD44+/EPCAM– cells; meanwhile, a slighter overexpression of the ALDH1A1 gene (fold change of 3) was observed for CD44+/EPCAM+ cells versus CD44–/EPCAM+ cells. Strong evidence also derived from the cytofluorimetric analysis of the ALDH^high^ sorted cells stained for CD44/EPCAM. Approximately 50% of the ALDH^high^ sorted cells were CD44+/EPCAM+, whereas only 2.6% of the ALDH^low^ sorted cells were CD44+/EPCAM+.

Taken together, the results of the present research highlight a strong similarity between ALDH^high^ and CD44+/EPCAM+ cells, as suggested by cytofluorimetric analyses, tumor sphere-forming assays, and RT-PCR experiments.

The identification of surface markers in lung cancer can contribute to personalized medicine. Although there is still uncertainty in the identification of cancer stem cells especially in lung, the scientific community have pointed out their important role in prognosis and recurrence [25]. Furthermore, the identification of these markers may be helpful for a better epidemiological stratification of oncological patients. Hence, the identification of cancer stem cell markers should be considered as a crucial step in the development of novel cancer-specific molecular targeted therapies [25].

Our experiments, performed as proof of concept, showed that the superficial marker CD44+/EPCAM+ detected about half of the ALDH^high^ cell population. These two populations seem to be very similar in terms of stemness gene expression as well as their capacity to make spheroids. In a recent study, MacDonagh et al. reported that ALDH1-positive cells can survive under cisplatin treatment [26]. This resistance to cancer treatments is daily confirmed in many oncologic patients. Although ALDH is nowadays considered an important marker for cancer stem cells as well as also for epithelial cancer cells [27] and that some clinical trials are running at the present time for analyzing ALDH-targeting treatments effects against cancer [28, 29], we strongly agree with the scientific community about the importance to identify markers, in order to develop target treatments against cancer stem cells [30–32]. In particular, with regards of our study, even if we hypothesized that the percentage of CD44+/EPCAM+ in ALDH^high^ cell population would be less than 50% in other patients, the similarity of an intracellular marker highlighting ALDH^high^ cell population with a superficial marker CD44+/EPCAM+ is a very important concept in terms of target treatment. In summary, our research is an important starting point for further studies that are needed to better define the CD44+/EPCAM+ superficial marker highlighting lung cancer stem cells.

### Limitations

A limitation of our study was that we did not perform formal sample size calculations for our main cytofluorimetric analysis. Nevertheless, the statistical power was calculated ex post and was satisfactory.

## MATERIALS AND METHODS

The study was conducted by analyzing the expression of ALDH^high/low^ cells and the positivity for CD44 and EPCAM antigens in a sample of surgical lung cancer tissue. The main approach was a cytofluorimetric analysis of ALDH expression and positivity for CD44/EPCAM on primary cell population obtained from 23 patients harboring NSCLC. Moreover, the following additional experiments were performed, on 1 patient each: 1) a cytofluorimetric analysis of CD44+/EPCAM+ on ALDH^high/low^ sorted cell subpopulations; 2) a tumor sphere-forming assay on CD44+/EPCAM+ sorted cells; 3) a real-time polymerase chain reaction (RT-PCR) for the ALDH1A1 mRNA expression on CD44+/EPCAM+ sorted cells.

### Study design and sample size

The present study was a cross-sectional study and was conducted in accordance with the Strengthening the Reporting of Observational Studies in Epidemiology (STROBE) guidelines [33].

The outcomes evaluated in the study were the expressions of ALDH^high^, CD44+/EPCAM+, CD44+/EPCAM–, and CD44–/EPCAM+ cells. The study primarily aimed at assessing the correlation and the pairwise difference between the expression of ALDH^high^ and CD44+/EPCAM+ cells. Secondarily, correlations between ALDH^high^ and CD44+/EPCAM– cells and between ALDH+ and CD44–/EPCAM+ cells were considered.

A formal calculation for sample size was not performed. A sample of patients who met the inclusion criteria during a 14-month timeframe was used. Statistical power calculations were performed ex post and were reported.

### Study population

Overall, 24 patients met our inclusion criteria within those harboring NSCLC undergone major lung resection by lateral thoracotomy at the Division of Thoracic Surgery of Modena University Hospital (Italy) for stage I, II, or IIIA non-small-cell lung cancer (8th tumor, node, metastasis (TNM) staging system) in the period from December 2017 to January 2019 were included in the study. A main cytofluorimetric analysis was performed in 23 patients; for one patient an additional cytofluorimetric analysis was added and in another one, the tumor sphere-forming assay was carried out. The last patient enrolled was only included for the additional RT-PCR experiment. Inclusion criteria were aged between 18 and 85, R0 resection, availability of formalin fixed, paraffin embedded surgery specimen from the primary tumor, and availability of fresh surgical specimen for cytofluorimetric analysis. Exclusion criteria were incomplete resection, unknown tumor, node, metastasis (TNM) status, synchronous tumors, and previous lung cancer.

### Primary cells isolated from human lung cancer

Tumor tissues were obtained within 1 to 2 h after surgical removal, washed in sterile Dulbecco’s phosphate-buffered saline (PBS) (L1825-BC – Merck Millipore, Italy), and mechanically minced into small pieces (2 to 4 mm). Minced samples were digested using a tumor dissociation kit in a disposable gentle MACS™ C-Tube (Miltenyi Biotec, Italy) according to the manufacturer’s instructions. Samples were digested for 60 min at 37°C in a gentle MACS Octo dissociator, filtered through 70-μm sterile cell strainers, centrifuged at 300 × g for 5 min, and resuspended in a DMEM and HAM’S F12 media mixture (2:1) (Gibco) containing 50 IU/mL penicillin-streptomycin and 4 mM glutamine. Viable cells were counted using an optic phase-contrast microscope [7].

### ALDEFLUOR assay

Single-cell suspensions of primary tumor cells were diluted in ALDEFLUOR assay buffer containing BODIPY-aminoacetaldehyde (STEMCELL Technologies, Vancouver, BC). The assay was performed according to the manufacturer’s protocol. Briefly, at least 5,000,000 tumor cells were resuspended in ALDEFLUOR BUFFER (5 μl/10^6^) and stained with ALDEFLUOR substrate. Immediately, 5 × 10^5^ cells were transferred to a control tube containing 5 μl of diethylaminobenzaldehyde (DEAB), which is a specific inhibitor of ALDH. Control and test samples were incubated for 45 min at 37°C and protected from light. Cells were centrifuged at 300 × g for 5 min. The cell pellet was resuspended in 1 ml of ALDEFLUOR assay buffer [7, 13].

### Cytofluorimetric analysis and cell sorting

Primary tumor cell suspensions were stained with ALDEFLUOR (Stem Cell) and allophycocyanin (APC)-conjugated anti-CD45 (Becton Dickinson, Franklin Lakes, NJ), phycoerythrin (PE)-conjugated anti-EPCAM (Becton Dickinson), and Brilliant Blue 515 (BB515) anti-CD44 (Becton Dickinson). An isotype control sample for each condition was used to exclude the autofluorescence background. Cell morphology was evaluated using side scatter (SSC) and forward scatter (FSC). Dead cells were excluded using 7-amino-actinomycin D (7-AAD) staining. Both types of cells, ALDH^high^ and double-positive CD44/EPCAM, were analyzed and sorted. The gate was set based on ALDH^high^ cells in 1 case and on CD45-negative-CD44+/EPCAM+, CD45-negative cells-CD44+/EPCAM– and CD45-negative cells-CD44–/EPCAM+ in the other cases.

Cell sorting was performed using a FACSAria III (Becton Dickinson). The results were analyzed using fluorescence-activated cell sorting (FACS) Diva software (Becton Dickinson, Franklin Lakes, NJ). Sorted cells were directly lysed for gene expression analyses and seeded in culture for tumor sphere assay.

### Tumor sphere-forming assay

A tumor sphere-forming assay on CD44+/EPCAM+ cells was performed, as previously assessed by our research group for ALDH^high^ cells [7]. The CD44+/EPCAM+ single-cell suspension was seeded in 24 ultralow attachment well plates. Cells were cultured in a mixture of serum-free Dulbecco’s modified Eagle’s medium (DMEM) and HAM’S F12 media (2:1) (Gibco) containing 50 IU/mL penicillin-streptomycin and 4 mM glutamine supplemented with 5 μg/ml insulin, 10 ng/ml epidermal growth factor (EGF), 20 ng/ml basic fibroblast growth factor (bFGF), 0.18 nM adenine, and 2 nM triiodotironin. The cells were cultured in 5% CO_2_ at 37°C for 3 weeks, and the media were replaced or supplemented with fresh growth factors twice per week. The entire well was digitally photographed using inverted phase-contrast microscopy (Zeiss Axioskop and Axiocam ICc3 color camera). All images were analyzed using the AxioVision software (Zeiss). The total number of spheres was counted, and the spheres’ areas were manually measured at 2, 7, and 21 days from seeding.

### Real-time PCR (RT-PCR)

Total cellular RNA was extracted from double-positive CD44/EPCAM sorted cells using the RNeasy Mini Kit (Qiagen) according to the manufacturer’s instructions. Total RNA (2 μg) was reverse transcribed using the RevertAid™ First Strand cDNA Synthesis Kit (Thermo Scientific). Following cDNA synthesis, real-time PCR (RT-PCR) was performed in technical replicates for each sample using FAST SYBR™ Green detection chemistry (Applied Biosystems) on Step One instrument. Human ALDH1A1 and glyceraldehyde 3-phosphate dehydrogenase (GAPDH) were amplified using gene-specific primers (ALDH1A1: forward primer 5′- TGTTAGCTGATGCCGACTTG-3′, reverse primer 5′-TTCTTAGCCCGCTCAACACT-3′; GAPDH: forward primer 5′-ACATCGCTCAGACACCATG-3′, reverse primer 5′TGTAGTTGAGGTCAATGAAGGG-3′). The forward and reverse primers were designed using IDT PrimerQuest1 (http://eu.idtdna.com/PrimerQuest/Home/Index). The cycling parameters consisted of denaturation at 95°C for 10 min and 40 cycles of 15 sec at 94°C, 30 sec at 60°C, and 1 min at 72°C, followed by a continuous melting curve.

### Statistical analysis

The continuous variables were described in terms of mean, standard deviation (SD), median, and range. The categorical variables were described as absolute and percentage frequencies.

Expression of all cells was measured as the percentages of 7-AAD negative (7-AAD-) cells. We calculated the correlations of the expression of ALDH^high^ cells with the expression of CD44+/EPCAM+, CD44+/EPCAM–, and CD44–/EPCAM+ cells. Both Pearson’s linear correlation and Spearman’s rank correlation coefficients were considered. The difference between the expression of ALDH^high^ cells and the expression of CD44+/EPCAM+ cells was assessed using a paired *t*-test. The statistical power of the test was calculated ex post based on the observed standard deviation of the mean differences and considering 3 target differences equal to 2%, 1.5%, and 1%. A linear regression model that considers ALDH^high^ cells expression as the dependent variable and CD44+/EPCAM+ cells expression as the independent variable was used to assess the linear relationship of the two cells subpopulations. The regression line was reported graphically.

RT-PCR data were analyzed according to the methods described in [34]. The relative mRNA expression of ALDH+ cells was reported as Delta Delta Ct (ΔΔCt) and as fold changes (FC, equal to 2^-ΔΔCt^). Two relative mRNA expressions of ALDH^high^ cells were calculated: 1) CD44+/EPCAM+ compared to CD44+/EPCAM– cells; 2) CD44–/EPCAM+ compared to CD44+/EPCAM+ cells.

The analyses were conducted using R 3.6.1 statistical software (The R Foundation for Statistical Computing, Wien) at the 95% confidence level.

## CONCLUSIONS

Our study is the first attempt toward the utility of a double superficial marker such as CD44+/EPCAM+ for the identification and further the targeting of lung cancer stem cells. This will be helpful for the setting of new treatments against lung cancer stem cells as well as also for a better control of the tumor growth.

## Consent for publication

This Study, involving human subjects, human material, and human data, has been performed in accordance with the Declaration of Helsinki and has been approved by the Ethics committee at University Hospital of Modena, MODENA, Italy, on 17 March 2017, Prot. N. 914/C.E. Further information and documentation to support this is available to the Editor on request. All the patients included in this Study have signed an informed consent before being enrolled. Further information and documentation to support this is available to the Editor on request. Consent for publication of data has been obtained from study participants. The datasets used and/or analyzed during the current study are available from the corresponding author on reasonable request.

## SUPPLEMENTARY MATERIALS



## Abbreviations

NSCLC: non-small cell lung cancer
ALDH: aldehyde dehydrogenase
CD44: cluster of differentiation 44
EPCAM: epithelial cell adhesion molecule
FACS: fluorescence-activated cell sorting
CSCs: cancer stem cells
SSC: side scatter
FSC: forward scattered
