# Supplementary material for: CD44+/EPCAM+ cells detect a subpopulation of ALDHhigh cells in human non-small cell lung cancer: A chance for targeting cancer stem cells?
Source: Oncotarget. 2020 Apr 28;11(17):1545–55. doi: 10.18632/oncotarget.27568 (PMC7197447; doi:10.18632/oncotarget.27568)
Supplement: Supplementary file 1 [file oncotarget-11-1545-s001.pdf]

CD44+/EPCAM+ cells detect a subpopulation of ALDH^{high} cells in human non-small cell lung cancer: A chance for targeting cancer stem cells?

SUPPLEMENTARY MATERIALS

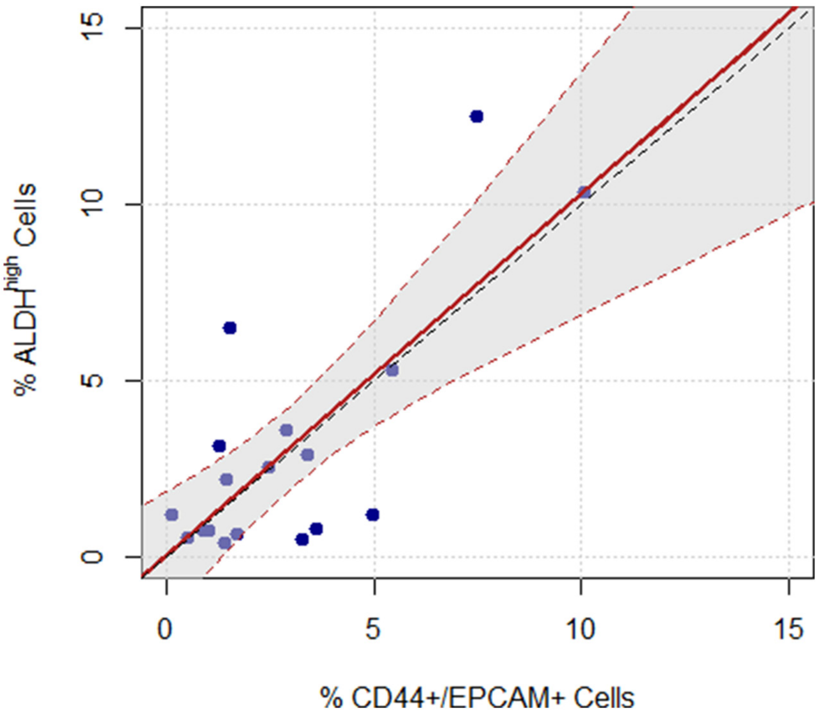

Supplementary Figure 1: Linear relation between the subgroup of ALDH^{high} and CD44+/EPCAM+ adenocarcinoma sorted cells. The red line represents the linear regression equation, considering adenocarcinoma ALDH^{high} as the dependent variable and adenocarcinoma CD44+/EPCAM+ as the independent variable. The shaded area represents the confidence interval for the regression equation.

Supplementary Table 1: Main results of the study in the subgroup of patients harboring adenocarcinoma

Cytofluorimetric analysis		All samples (*n* = 18)
ALDH+ (% on 7AAD- cells)	mean ± SD	3.1 ± 3.5%
	median (range)	1.7% (0.4%; 12.5%)
CD44+/EPCAM+ (% on 7AAD- cells)	mean ± SD	3.0 ± 2.6%
	median (range)	2.1% (0.1%; 10.1%)
Δ ($ALDH^{high}$ minus CD44+/EPCAM+)	mean ± SD	0.1 ± 2.5%
	median (range)	0.0% (–3.8; 5.0%)
	95% CI	–1.0; 1.3%
	p-value	0.8191
Pearson's correlation ($ALDH^{high}$ and CD44+/EPCAM+)	r (95% CI)	0.76 (0.46; 0.91)
	p-value	0.0002
Spearman's correlation ($ALDH^{high}$ and CD44+/EPCAM+)	r	0.51
	p-value	0.0314

SD = standard deviation; r = correlation coefficient; 95% CI = 95% confidence interval.
